# Bortezomib, Bendamustine and Dexamethasone vs Thalidomide, Bendamustine and Dexamethasone in Myeloma patients presenting with renal failure (OPTIMAL): a randomised, multi-centre phase II trial

**DOI:** 10.1038/s41408-022-00758-7

**Published:** 2022-11-29

**Authors:** Karthik Ramasamy, Gulnaz Iqbal, Richard Brouwer, Victoria Stalker, Salma Akhtar, Sherin Varghese, Jindriska Lindsay, Stephen Schey, Mark Drayson, Janet Dunn

**Affiliations:** 1 Churchill Hospital, Oxford University Hospitals NHS Foundation Trust, Oxford, UK; 2 Radcliffe department of Medicine, Oxford University, Oxford, UK; 3 Oxford Translational Myeloma Centre, Oxford, UK; 4 Warwick Clinical Trials Unit, University of Warwick, Coventry, UK; 5 East Kent Hospitals University Trust, Canterbury, Kent UK; 6 King’s College Hospital NHS Foundation Trust, London, UK; 7 Division of Immunity & Immunotherapy, University of Birmingham, Birmingham, UK

**Keywords:** Cancer therapy, Randomized controlled trials, Myeloma

Dear Editor,

Renal impairment is a serious but reversible complication of multiple myeloma (MM). Up to 20–25% of patients will have severe renal impairment at initial diagnosis [1–5], and it occurs in up to 50% of patients at some stage during their disease [6]. It is possible to reverse renal insufficiency in approximately half of patients [7] at diagnosis, but half will have some degree of persistent renal impairment, and of these 2–12% will require renal replacement therapy.

Over the past decade, overall survival prospects for patients with MM have considerably improved but less so for MM patients complicated by renal impairment. Recipients of dialysis and a diagnosis of MM have median overall survival of 2–3 years even in the novel agent induction era [8]. This is mainly because of a high early death rate, with 28% of newly diagnosed myeloma patients in myeloma trials with renal failure not surviving beyond 100 days compared with 10% overall [4].

Outcomes in patient’s presenting with raised serum free light chain levels (sFLC) and renal impairment correlate with the speed of reduction in sFLC. The MERIT trial, showed patients who were alive and dialysis free at 100 days (as compared to those dead or on dialysis) had lower levels of FLC at entry and greater reductions in sFLC in the first two weeks [9].

Bortezomib-based induction therapy has been given a Grade A recommendation by IMWG consensus, primarily based on retrospective cohort studies of patients with renal impairment and non-renal clearance of this agent [10]. Thalidomide-based therapy is also non-renally excreted in contrast with largely renal elimination of an alternative IMiD, Lenalidomide and given a Grade B recommendation by IMWG. No studies in myeloma have compared by randomised controlled trial (RCT) Bortezomib with Thalidomide in an induction setting, for newly diagnosed myeloma with or without renal impairment.

Bendamustine has dual mechanism of action as an alkylating agent and antimetabolite [11], exhibits partial cross resistance to other alkylating agents, and is effective in patients with relapsed myeloma. Up to 20% of administered bendamustine is renally eliminated within 24 h making it a potential therapeutic option for patients with renal impairment. Bendamustine and bortezomib in combination with steroids have been used both in newly diagnosed myeloma patients with renal impairment and in relapsed refractory myeloma patients [12].

Thus, there is rationale for comparing in an RCT the effectiveness of a thalidomide versus bortezomib in a dexamethasone and bendamustine regimen on the basis that both are effective and can be given to patients even with advanced renal impairment.

We hypothesised a significant difference in achieving a reduction in sFLC during the first two cycles of therapy when comparing thalidomide versus bortezomib in combination with bendamustine and dexamethasone, which would lead to an observed renal response. In addition, sFLC response at the end of two cycles would allow for early identification of poor responders who may benefit from alternative therapy.

OPTIMAL was a two-arm, phase II, multi-centre RCT. Patients at least 18 years of age with newly diagnosed symptomatic myeloma and renal impairment (GFR < 30mls/min) were eligible for the study.

Participants were randomised to receive an average of 4 cycles of either Bortezomib (Arm A) or Thalidomide (Arm B); all participants received Bendamustine 60 mg/m^2^ intravenously on days 1 and 8 of each cycle and Dexamethasone 40 mg orally days 1–2, 4–5, 8–9 and 11–12 of each cycle in 3-week cycles. Participants in Arm A received Bortezomib 1.3 mg/m^2^ subcutaneously on days 1, 4, 8, and 11 of each cycle whilst those in Arm B received Thalidomide at 100 mg once daily orally on days 1–21. The treatment period for participants receiving 4 cycles of therapy was 12 weeks. Participants not considered suitable for autologous stem cell transplant were given up to 6 cycles.

Assessments performed during treatment included central and local laboratory evaluations, concomitant medications, significant toxicity, and adverse events. Sampling was performed at the end of each treatment cycle (weeks 1–9 and 12).

All participants receiving at least 2 cycles of treatment were followed up. Assessments and sampling were performed at 1 month post treatment follow-up and end of treatment. Data was then requested at 12 months post-randomisation. Participants were also asked to complete an EQ-5D-3L questionnaire.

The primary outcomes were the proportion of participants with sFLC response defined as >50% reduction after two cycles of trial therapy (Modified IMWG Uniform Criteria of Response and Progression, 2011) and renal response at end of 4 cycles of therapy (IMWG renal response criteria [13]). Secondary outcomes assessed included the renal response at the end of 2 cycles of therapy, overall survival, toxicity and haematological toxicity, quality of life (QoL) and sFLC response at end of weeks 1–6, 9, and 12 of treatment.

Optimal screened 88 patients for the trial between 2014 and 2019, of which 31 patients were randomised to receive BBD (16 patients) or BTD (15 patients). Of the 57 screen failures, 10 were due to the patient being too frail and 8 due to renal function improvement. Other reasons for failure include clinical ineligibility, offered different treatment, and living out of area. Patient characteristics were balanced across treatment groups, in terms of stratification variables and baseline clinical assessment (Table 1 and Fig. S1). The primary endpoint of sFLC response was assessed in 30 patients where samples were available at screening and the end of two cycles of trial treatment. Data suggests a significant benefit for treatment with BBD, with 13 patients on BBD arm achieving vGPR after receiving two cycles of treatment compared to three patients on the BTD arm, *p* = 0.003, Table 2. Renal response after four cycles, was assessed for 20 patients and did not differ significantly between the two arms, *p* = 0.12, Table 2. However, five patients receiving Bortezomib achieved complete or partial renal response compared to one patient on Thalidomide. Analysis of renal response using eGFR alone, assessing for an increase in eGFR >25%, confirmed renal function did not differ between the trial arms, *p* > 0.99. Nine patients were on dialysis at the time of screening (6 on BBD and 3 on BTD) and continued to require dialysis after two cycles. Renal response after 2 cycles, was assessed in 28 patients. Renal function based on IMWG renal response criteria did not differ between the trial arms, *p* = 0.45. Analysis of renal response using eGFR alone confirmed renal function did not differ between the trial arms, *p* > 0.99.

**Table 1:** Patient characteristics by treatment arm.

Factor	Grouping	BBD (*n* = 16)		BTD (*n* = 15)		Total (*n* = 31)	
		*n*	%	*n*	%	*n*	%
Age (yrs)^a^	≤70 yrs	8	50	8	53	16	52
	>70 yrs	8	50	7	47	15	48
CKD stage^a^	4	6	38	5	33	11	35
	5	10	62	10	67	20	65
Sex	Male	8	50	9	60	17	55
	Female	8	50	6	40	14	45
ECOG at screening	0	2	13	5	33	7	23
	1	8	50	8	53	16	52
	2	3	19	1	7	4	13
	3	3	19	0	0	3	10
	4	0	0	1	7	1	3
Intention to perform aSCT	No	9	56	7	47	16	52
	Yes	7	44	8	53	15	48
On dialysis	No	10	63	12	80	22	71
	Yes	6	37	3	20	9	29
ISS stage	I	0	0	0	0	0	0
	II	0	0	1	7	1	3
	III	14	87	14	93	28	90
	Missing	2	13	0	0	2	6
Pre-existing condition that	No	14	87	14	93	28	90
may cause renal damage	Yes	2	13	1	7	3	10
	If yes, eGFR decline?	1	-	1	-	2	-
Evidence of bone disease	No	6	37	5	33	11	35
	Yes	8	50	9	60	17	55
	Missing	2	13	1	7	3	10
Type of bone disease	Vertebral fractures	3	20	4	27	7	28
	Lytic lesions	6	38	6	40	12	48
	Fractured rib	1	6	1	7	2	8
	Osteoporosis/osteopenia	2	13	1	7	3	12
	Clavicle	1	7	0	0	1	4
ECG normal	No	4	25	3	20	7	23
	Yes	12	75	12	80	24	77
	Missing						
Peripheral neuropathy	No	12	75	10	67	22	71
	Yes	3	19	3	20	6	19
	Missing	1	6	2	13	3	10

^a^Stratification factor.

**Table 2:** Primary outcomes sFLC response to two cycles of therapy, renal response to 4 cycles of therapy, and Quality of Life (EQ-5D-3L index and EQ VAS scores) by treatment arm.

Outcome	Response depth	BBD (*n* = 16)		BTD (*n* = 14)		Total (*n* = 30)		Fisher’s
		*N*	%	*N*	%	*N*	%	*P*
sFLC response	vGPR	13	81	3	22	16	54	0.003
After 2 cycles	PR	2	13	8	57	10	33	
	MR	0	0	1	7	1	3	
	SD	1	6	1	7	2	7	
	PD	0	0	1	7	1	3	
Renal response	CR/PR	5	31	1	7	6	19	0.12
After 4 cycles	MR	3	19	7	47	10	32	
	No response	3	19	1	7	4	13	
**QoL**	**Timepoint**	**BBD**			**BTD**			**T-Test p**
		***N***	**Mean**	**SD**	***N***	**Mean**	**SD**	
EQ-5D score	Baseline	8	0.72	0.15	9	0.69	0.35	
	1 month FU	8	0.69	0.19	9	0.80	0.28	
	Change	8	−0.04	0.19	9	0.11	0.39	0.33
EQ VAS score	Baseline	8	59	19	8	59	19	
	1 month FU	8	69	19	9	84	15	
	Change	8	10	21	8	13	13	0.72

The EQ-5D descriptive system comprises the following five dimensions: mobility, self-care, usual activities, pain/discomfort and anxiety/depression. Each dimension is scored on a scale of 1 to 3. Higher score equates to a worse outcome.

*vGPR* very good partial response, *PR* partial response, *MR* minimal response, *SD* stable disease, *PD* progressive disease, *CR* complete response.

Two haematological toxicity-related SAEs were reported on BBD (Table S1). In addition, nine haematological AEs were also recorded (3 on BBD and 6 on BTD). No statistically significant differences were detected between SAEs or AEs by treatment arm, Fisher’s Exact *p* = 0.48 and *p* = 0.25, respectively although the study was underpowered to detect such differences.

Nine deaths were reported (7 on BBD and 2 on BTD; Table S2). No difference in overall survival was observed between treatment arms, *p* = 0.25 (Fig. S2) but this was a small study with a shorter follow up and was not powered to show a survival difference.

Although no differences were detected in QoL between baseline and one month follow-up between treatment arms, age group or dialysis status this may be due to the small patient numbers and early assessment timepoints and should be explored further in future studies. Improvement in anxiety and depression was observed overall with 59% of patients reporting none at baseline increasing to 76% at follow up (Table 2).

To conclude, this study examined the differences between two combination chemotherapeutic interventions and their ability to induce a deep myeloma response. Deeper myeloma responses are more durable and are associated with improvement in renal function in previous single arm retrospective studies and the prospective MYRE trial, where early reduction of sFLCs was a predictor of dialysis independence [14]. Patients treated with Bortezomib had a significantly higher response rate in comparison with Thalidomide. Deeper response did not translate into improved renal response in the Bortezomib arm at the end of induction therapy. The goal of therapy in newly diagnosed myeloma patients with renal failure is to induce significant depth of haematological response and induce improvement in renal function aligned to improvement in QoL. This study shows a Bortezomib-based approach has a better chance of inducing such a response.

This is the first prospective study testing the IMWG renal response criteria. It remains unclear as to whether it is sensitive enough to pick up an improvement in renal function in patients with renal impairment. Additional confounding factors include other causes of renal impairment in patients such as diabetes, hypertension, reno vascular disease, age, and use of nephrotoxic agents.

This is the first RCT to explore two different treatment combinations in myeloma patients with renal impairment. Recently the EULITE [15] and mYRE [14] RCTs examined the utility of removing sFLC by extended high cut off haemodialysis in myeloma patients with haemodialysis dependent renal failure receiving Bortezomib based combination induction therapies. These RCTs showed no significant benefit to the removal of sFLC by high cut off haemodialysis. This could be due to the deep response (reduced secretion of sFLC) achieved by induction therapy and thus physical removal of sFLC contributing limited value to improving renal outcomes. Our study adds support to the EULITE and mYRE trials suggesting that Bortezomib-based induction should be standard of care frontline therapy for myeloma patients. The small numbers do not allow us to validate the ability of IMWG renal response criteria to predict differences in renal outcomes.

Further research should build on the Bortezomib based combination therapy which was superior as induction chemotherapy for newly diagnosed myeloma patients with renal failure. Despite deep responses, a number of patients progressed early (<1 year) following discontinuation of induction therapy. One potential approach to limit this is exploring continuous therapy approaches which is increasingly becoming standard of care in myeloma. The addition of more recently available immunotherapeutic agents such as daratumumab and isatuximab which can be safely used in renal impairment may further improve outcomes.

## Supplementary information

Supplemental Figures S1 and S2

Table S1: Toxicity events by treatment for BBD and BTD

Table S2: Cause of deaths

## Data Availability

The datasets generated during and/or analysed during the current study are available from the corresponding author on reasonable request.
