# Supplementary material for: Bortezomib, Bendamustine and Dexamethasone vs Thalidomide, Bendamustine and Dexamethasone in Myeloma patients presenting with renal failure (OPTIMAL): a randomised, multi-centre phase II trial
Source: Blood Cancer J. 2022 Nov 29;12(11):162. doi: 10.1038/s41408-022-00758-7 (PMC9708638; doi:10.1038/s41408-022-00758-7)
Supplement: Supplementary file 1 — Supplemental Figures S1 and S2 [file 41408_2022_758_MOESM1_ESM.pdf]

Figure S1: Consolidated Standards of Reporting Trials (CONSORT) Chart of OPTIMAL trial

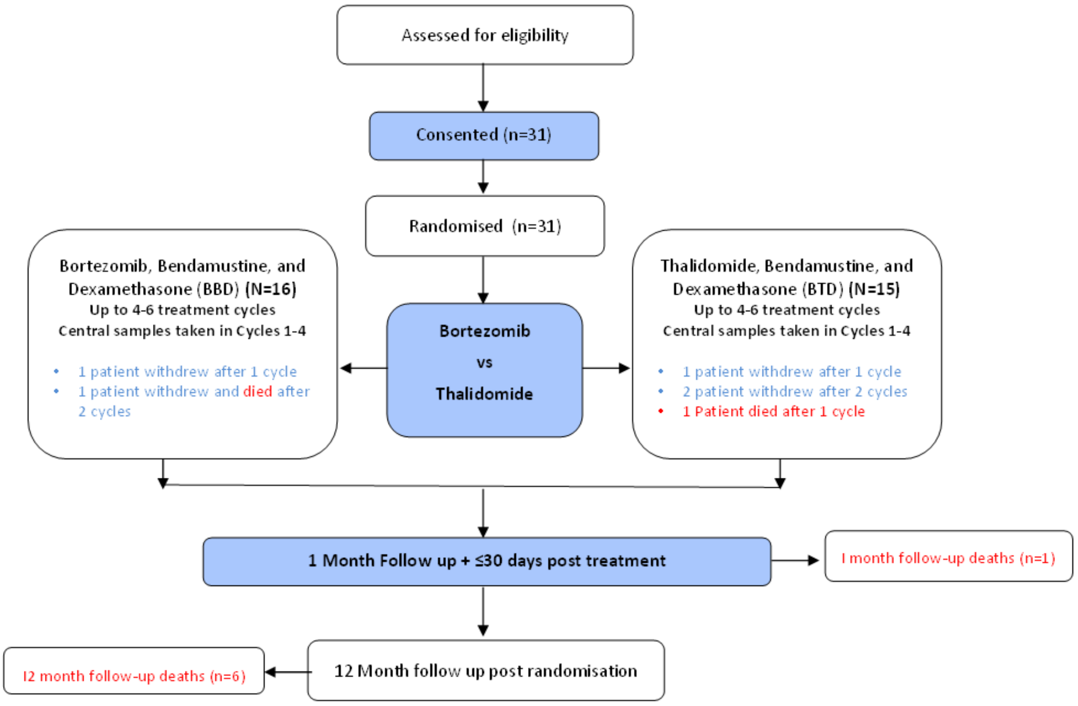

Figure S2: Overall survival by treatment arm

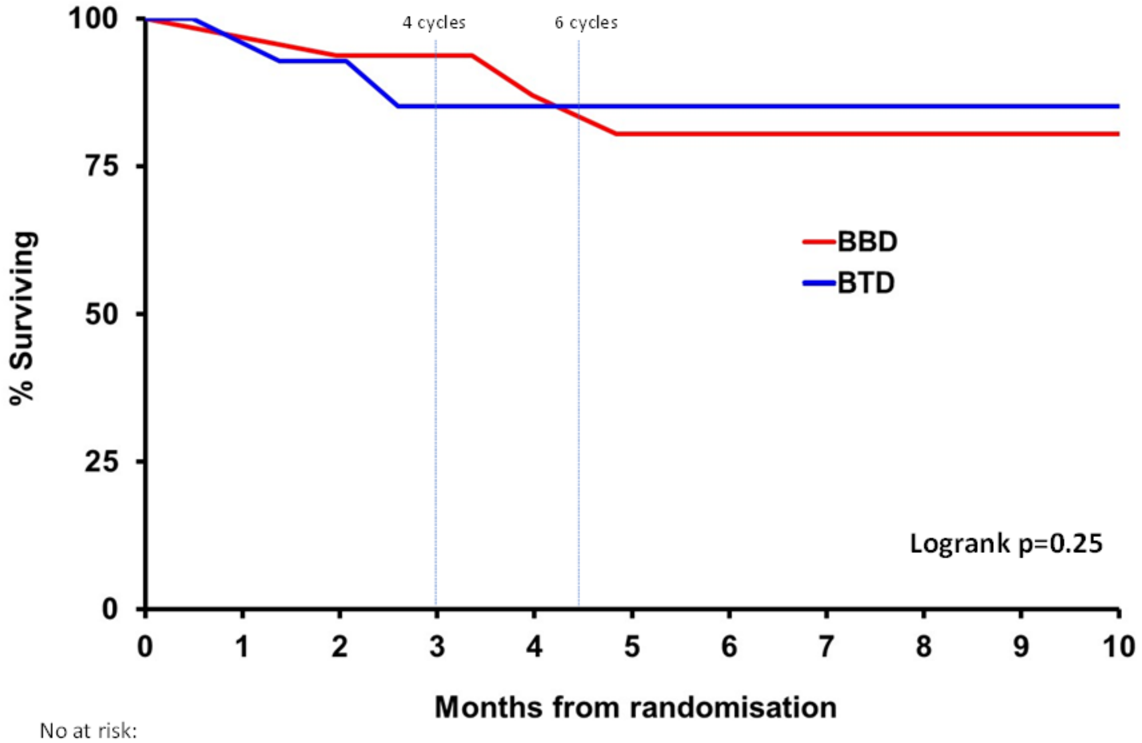

No at risk:

BBD	16	16	15	15	13	12	12	12	12	12	12
BTD	15	14	13	11	11	11	10	10	10	10	10
